# Lipid Metabolism in Pregnancy Women with Hypothyroidism and Potential Influence on Pregnancy Outcome

**DOI:** 10.1155/2024/5589492

**Published:** 2024-07-09

**Authors:** Yuxin Qin, Ying Wu, Huanhuan Zang, Xiangguo Cong, Qiong Shen, Lei Chen, Xinxin Chen

**Affiliations:** Department of Endocrinology The Affiliated Suzhou Hospital of Nanjing Medical University, 26 Daoqian Road, Suzhou 215000, China

## Abstract

Thyroid hormone (TH) is essential for maintaining normal physiological processes during pregnancy, including the metabolism of energy materials in both the mother and fetus and the growth and development of fetal bone and nervous system. TH can act on the liver, fat, and other tissues and organs to participate in lipid synthesis and breakdown through multiple pathways. Consequently, abnormal thyroid function is often accompanied by lipid metabolism disorders. Both clinical and subclinical hypothyroidism, as well as dyslipidemia during pregnancy, have been shown to be associated with an increased risk of multiple adverse pregnancy outcomes. Recently, there has been an increased interest in studying the alteration of lipidomic and hypothyroidism (both clinical and subclinical hypothyroidism) during pregnancy. Studies have suggested that altered lipid molecules might be used as potential biomarker and associated with adverse maternal and neonatal outcome. Thus, we summarized the associations between lipid metabolism and clinical or subclinical hypothyroidism during pregnancy in this review. Then, we discussed the underlying mechanisms of thyroid dysfunction and lipid metabolism. In addition, we reviewed the possible effect of dyslipidemia on pregnancy and neonatal outcome. However, the relationship between hypothyroidism during pregnancy and changes in the lipid profile and how to intervene in the occurrence and development of adverse pregnancy outcomes require further study.

## 1. Introduction

Thyroid disease is the most prevalent endocrine disorder during pregnancy [1]. Thyroid hormones are critical for the metabolism of carbohydrates, calcium, phosphorus, fat, and other energy substances in both pregnant women and their developing fetuses. The fetal thyroid gland begins to function at approximately 20 weeks of gestation, but it is not fully functional until after birth. Therefore, there is an increased physiological demand for thyroid hormones during early pregnancy. Any disruption in maternal thyroid function can potentially affect the fetus, leading to developmental issues and an increased risk of adverse pregnancy outcomes. Additionally, thyroid hormones are instrumental in fostering the growth and development of fetal bones and reproductive organs, playing a vital role in ensuring normal fetal development and maturation. Pregnant women with clinical or subclinical hypothyroidism are at risk of experiencing a range of maternal and fetal complications, including miscarriage, premature delivery, placental abruption, preeclampsia, gestational diabetes, and impaired neurodevelopment of the offspring [2, 3].

Thyroid dysfunction has been indicated as an independent risk factor for dyslipidemia [4, 5]. Thyroid hormones significantly influence various aspects of lipid metabolism, as they can enhance the utilization of lipid substrates, mobilize triglycerides (TGs) stored in adipose tissue, and increase the activities of hepatic lipase (HL) and cholesterol transfer protein (CETP). Disorders of maternal lipid metabolism have been associated with an increased risk of gestational diabetes, gestational hypertension, preeclampsia, and preterm birth [6–8]. The interest in studying possible associations between thyroid dysfunction and lipidemia during pregnancy has increased. It was suggested that altered lipid molecules in pregnant women with hypothyroidism might be used as potential biomarker and associated with adverse maternal and neonatal outcome.

Here, we aim to provide a comprehensive review of the association between clinical or subclinical hypothyroidism and dyslipidemia during pregnancy. In addition, we want to emphasize the relationship between dyslipidemia and an increased risk of adverse outcomes in pregnant women with thyroid disease and examine the possible underlying mechanisms.

### 1.1. Physiological Changes in Lipids during Pregnancy

**Figure 1:**
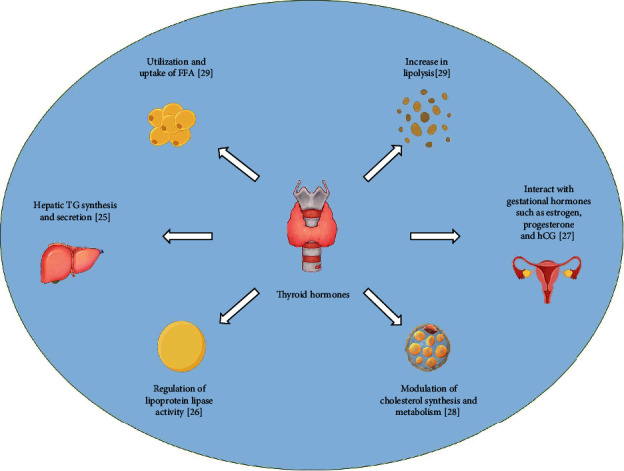
The scheme outlines the physical action of thyroid hormones on lipid metabolism during pregnancy. FFA: free fatty acid; hCG: human chorionic gonadotropin.

To meet fetal growth and development needs, physiological changes in lipid metabolism occur during normal pregnancy (as shown in Figure 1). Changes in blood lipids during normal pregnancy have been reported to be associated with fluctuations in sex hormones during pregnancy. The first and second trimesters are characterized by high progesterone levels, which promote anabolic processes and are mediated by progressive increases in insulin levels [9]. The third trimester is characterized by high estrogen levels, which shift metabolism toward catabolism [10].

One of the key lipid changes during pregnancy is an increase in total cholesterol and TG levels. This is primarily due to the secretion of lipoproteins by the placenta, which is responsible for transporting lipids to the fetus. Additionally, the production of estrogen and progesterone, which are essential for maintaining pregnancy, is associated with an increase in the levels of these hormones in the mother's bloodstream. Another important lipid change is a decrease in high-density lipoprotein cholesterol (HDL-c), which is often referred to as “good” cholesterol. This decrease is thought to be related to increased estrogen levels. Estrogen can downregulate the expression of HDL receptors, resulting in reduced HDL-c levels. Furthermore, pregnancy is also associated with increased levels of low-density lipoprotein cholesterol (LDL-c), which is often referred to as “bad” cholesterol. The upregulation of maternal lipolysis mediated by estrogen increases the production of very low-density lipoprotein (VLDL), which subsequently stimulates the transplacental transport of lipids. The levels of apolipoprotein B (Apo-B) are significantly lower in the first trimester and return to prepregnancy levels in the second trimester. In the third trimester, Apo-B levels slightly increase [11].

These changes support the increased energy demands of pregnancy and the growth requirements of the fetus. The blood lipid levels of normal pregnant women begin to increase at 9-13 weeks of pregnancy and continue to increase gradually with increasing gestational age. Most studies suggest that the increase in blood lipids occurs primarily in the second trimester. However, a cross-sectional study of serum cholesterol in healthy singleton pregnant women revealed that the median values of TC and VLDL increased throughout gestation and were greater than the nonpregnant adult range in the first trimester [12]. Blood lipid levels peaked at 31-36 weeks of gestation and then remained at high levels. Physiological changes in lipid metabolism are generally temporary and resolve after pregnancy [13].

### 1.2. Clinical or Subclinical Hypothyroidism and Dyslipidemia

**Figure 2:**
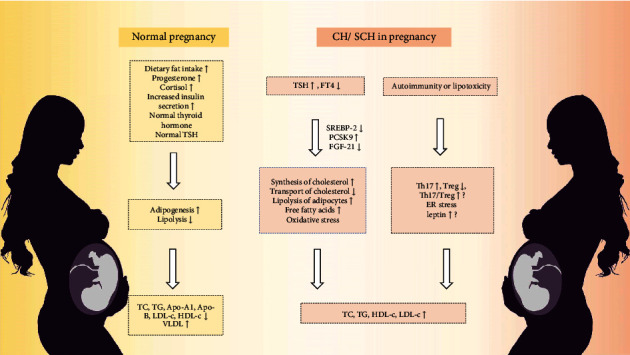
The comparison of lipid changes in healthy pregnant women and pregnant with thyroid disease. CH: clinical hypothyroidism; SCH: subclinical hypothyroidism; TSH: thyroid-stimulating hormone; FT4: free thyroxine; SREBP: sterol regulatory element-binding protein; FGF: fibroblast growth factor; PCSK9: proprotein convertase subtilisin 9; TC: total cholesterol; TGs: triglycerides; HDL-c: high-density lipoprotein cholesterol; Apo-A1: apolipoprotein A1; Apo-B: apolipoprotein B; LDL-c: low-density lipoprotein cholesterol; VLDL: very low-density lipoprotein; Th17 cells: T helper 17 cells; Treg cells: regulatory T cells.

Hypothyroidism during pregnancy, defined by elevated thyroid-stimulating hormone (TSH) levels and reduced serum free thyroxine (FT4) levels, is a relatively common condition that affects 3-5% of pregnant women [14]. Several studies have examined lipid changes in pregnant women with thyroid dysfunction (as shown in Figure 2). One study of 1650 pregnant women with thyroid dysfunction revealed that those with clinical hypothyroidism exhibited higher levels of apolipoprotein A1 (Apo-A1) and Apo-B than those with normal thyroid function. Furthermore, TC levels were found to be positively correlated with TSH levels and inversely correlated with FT4 levels [15]. Another study by Xu and Zhong revealed that both TG and LDL-c levels were greater in the hypothyroidism group than in the subclinical hypothyroidism group and euthyroid group [16]. Zhou et al. also observed significantly elevated plasma TC and LDL-c levels in pregnant women with hypothyroidism compared to those with prepregnancy hypothyroidism, with evidence of hepatic TG accumulation [17].

Subclinical hypothyroidism (SCH) during pregnancy is defined by TSH levels above the upper limit of the pregnancy-specific reference range, while FT4 levels remain within the normal range. SCH is estimated to affect approximately 10% of pregnant women [15]. SCH is also associated with alterations in lipid profiles, although the extent and impact of these changes are still under investigation (as shown in Figure 2). Observational studies have shown that SCH patients in early pregnancy have TC, TG, HDL-c, and LDL-c levels than normal pregnant women [18]. In the second and third trimesters, SCH patients continued to exhibit higher TC, TG, and LDL-c levels, but there was no significant difference in HDL-c levels [19, 20]. Studies have revealed that SCH patients have altered serum fatty acid concentrations, with some fatty acids being more abundant in SCH patients in the second trimester than in normal women [21].

### 1.3. Lipidomic Study of Clinical or Subclinical Hypothyroidism in Pregnant Women

Lipidomic study represents an emerging discipline that holds great potential in revealing the association between lipid biology and disease. Lipids are essential molecules in organisms, playing critical roles in processes such as the structure of cell membranes, energy storage, signaling pathways, and inflammation. Through lipidomic study, scientists can gain a more comprehensive understanding of the changes in lipids under both healthy and disease states, thereby providing new insights and methods for the diagnosis, treatment, and prevention of diseases. Advanced techniques such as liquid chromatography–mass spectrometry (LC–MS) have revealed significantly different lipid metabolic patterns in SCH and clinical hypothyroidism (CH) groups compared to healthy control groups, with similar alterations observed in the SCH and CH groups. Shao et al. also found that lysophosphatidylcholine (LPC) (18 : 0) and LPC (20 : 0) levels were elevated in patients with hypothyroidism and found no significant difference in lipid metabolism between SCH patients and CH patients [22]. Cai et al. identified 10 differentially abundant metabolites that could serve as characteristic metabolites for patients with hypothyroidism during pregnancy, including increased levels of phosphatidylcholine (PC) (36 : 2) and phosphatidylethanolamine (PE) (36 : 4) and decreased levels of sphingomyelin (SM) (d42:6), SM (d42:7), and LPC (18 : 0) [23]. Li et al. reported that SM and PE levels were greater in a subclinical hypothyroidism group than in a control group [24]. Certain lipids, such as LPC, SM, PC, and PE, exhibited similar patterns in pregnant SCH and CH patients. This similarity in lipid profiles suggests a complex relationship among thyroid function, lipid metabolism, and pregnancy.

### 1.4. The Possible Effect of Thyroid Hormone on Lipid Metabolism

#### 1.4.1. Direct Effect of Thyroid Hormone on Lipid Metabolism

The dyslipidemia observed in patients with hypothyroidism during pregnancy is primarily related to a decrease in TH levels. First, TH influences fat synthesis, transportation, and degradation. TH increases the activity of CETP, which transfers cholesterol from HDL-c to LDL-c and VLDL [4]. TH also increases the activity of lipoprotein lipase, which hydrolyzes TG-rich lipoproteins and accelerates the transfer of cholesteryl esters from these lipoproteins to HDL-c, thereby lowering TG levels. A decrease in TH in hypothyroidism patients leads to a decrease in these effects, thus promoting an increase in serum TG and HDL-c levels. Second, TH upregulates the expression of LDL receptor messenger RNA (mRNA) on the liver cell membrane, which leads to an increase in LDL receptor uptake and a decrease in circulating cholesterol. The number of LDL receptors is reduced in hypothyroidism by promoting the occurrence of oxidative stress disorder in vivo; thus, LDL-c uptake is reduced. Third, hypothyroidism increases hepatic free fatty acid influx. This increases the synthesis of LDL-c in the liver and eventually leads to an increase in TG and LDL-c content [25, 26]. In addition, hypothyroidism during pregnancy may increase plasma TC and LDL-c more effectively than prepregnancy hypothyroidism. This may be because hypothyroidism during pregnancy under the influence of progesterone reduces hepatic cholesterol uptake more effectively than prepregnancy hypothyroidism by decreasing the number and activity of hepatic LDL receptors (LDLR) [27].

TSH is known to have lipolytic effects, particularly during the neonatal period, and its role in lipid metabolism may extend beyond the thyroid gland [21]. Thyroid-stimulating hormone receptor (TSHR) expression has been detected in various cell types, including lymphocytes, adipocytes, and hepatocytes, suggesting that TSH may regulate lipid metabolism through these nonthyroidal cells. In hepatocytes, TSH can upregulate the expression of hydroxy methylglutaryl coenzyme A reductase (HMGCR), the rate-limiting enzyme in cholesterol synthesis, via the cAMP/PKA/CREB signaling system [28]. Furthermore, TSH increases serum-free fatty acid (FFA) levels by stimulating lipolysis in adipocytes and phosphorylating lipids and hormone-sensitive lipases [29]. Animal studies have demonstrated that TSHR knockout mice have lower liver cholesterol and TG levels than wild-type mice, suggesting that TSH plays a role in lipid metabolism beyond the thyroid [30]. Thus, SCH during pregnancy is associated with dyslipidemia, which may be influenced by the lipolytic effects of TSH and alterations in lipid metabolism.

#### 1.4.2. Indirect Effect of Thyroid Hormone on Lipid Metabolism

Hypothyroidism can also lead to dyslipidemia by influencing regulatory factors such as sterol regulatory element binding proteins (SREBPs), proprotein convertase subtilisin/kexin type 9 (PCSK9), and fibroblast growth factor 19/21 (FGF19/21), which were reported to be associated with lipid metabolism.

*(1) Sterol Regulatory Element-Binding Protein (SREBP)*. Sterol regulatory element-binding protein (SREBP) plays an important role in lipid metabolism, especially in cholesterol metabolism. Studies show that TH boosts the expression of the SREBP-2 gene, which then activates the HMGCR gene, a key enzyme in cholesterol synthesis [31, 32]. SREBP-2 also binds to specific DNA sequences on the LDLR gene, enhancing its transcription and leading to more LDLR and increased clearance of LDL-c [33]. TH can affect the intracellular metabolism of acetyl-CoA carboxylase and fatty acid synthase after binding to SREBP-1C/carbohydrate response element-binding protein (ChREBP) [34]. In adipose cells, TSH can act on SREBP-2 to increase HMGCR mRNA level and promote lipolysis [35]. TSH also activates signaling pathways, such as PI3K/AKT/SREBP-2 and SREBP-2/HNF4/cholesterol 7*α*-hydroxylase, which can inhibit bile acid production in the liver through the TSHR [36]. This, in turn, affects the clearance of LDL-c.

*(2) Fibroblast Growth Factor*. The fibroblast growth factor (FGF) family includes 22 proteins with similar structures, and each member plays a role in various metabolic processes in mammals [37]. FGF21, primarily secreted by liver cells, is an important regulator of glucose and lipid metabolism [38]. Studies have shown that circulating FGF-21 levels are significantly reduced in patients with hypothyroidism [39]. TH has been shown to interact with FGF-21 gene to upregulate FGF-21 expression, thereby downregulating serum FFA concentration by inhibiting lipolysis in white adipose tissue (WAT) and activating FFAs uptake in WAT [40]. Adams et al. found that TH could enhance the expression of FGF-21 in mouse liver and, thus, promote *β*-oxidation [41]. Another study showed that the administration of FGF-21 peripherally could decrease the levels of circulating TH [42]. It has been found that FGF-21 secretion is increased in the liver of female mice in the third trimester [43]. Therefore, it is speculated that FGF-21 is involved in the physiological changes of blood lipids during pregnancy.

FGF19, secreted by the ileum, is involved in the negative feedback regulation of bile acid synthesis [44]. Serum FGF-19 levels were increased in patients with hypothyroidism and were correlated with circulating TSH levels [45]. Other studies have found that SREBP can downregulate the expression level of FGF-19 gene [46]. However, the underlying mechanism by which FGF-19 induces hyperlipidemia in patients with hypothyroidism is not yet fully understood.

*(3) Proprotein Convertase Subtilisin/Kexin Type 9*. Proprotein convertase subtilisin 9 (PCSK9) is an enzyme that binds to the LDLR on the surface of liver cells. TH could downregulate the serum PCSK9 levels through SREBP-1C and SREBP-2 [47]. In patients with hypothyroidism, there is a reduction in the expression of LDLR as well as the increase of LDL-c levels [48]. Kwakernaak et al. showed that TSH levels were positively correlated with serum PCSK9 levels even in the normal range of thyroid function, which partly depends on SREBP-1C, SREBP-2, and HMGCR [49]. Notably, during normal pregnancy, PCSK9 and ChREBP levels exhibit physiological elevations, and these alterations are particularly pronounced in obese individuals [50, 51].

*(4) Angiogenin-Like Proteins (ANGPTLs)*. Angiogenin-like proteins (ANGPTLs), especially ANGPTL3 and ANGPTL4, are known to play a role in regulating lipid metabolism by inhibiting lipases. A study by Yang et al. found that patients with hypothyroidism had significantly higher levels of circulating ANGPTL3, which was positively correlated with serum HDL-c levels and negatively correlated with serum T3 concentration [52]. Garces et al. found that compared with healthy nonpregnant and postpartum women, serum ANGPTL3 levels were significantly lower in healthy pregnant women from the first trimester to the third trimester. It was speculated that serum ANGPTL3 may be involved in the changes of lipid metabolism in the anabolic and catabolic stages of pregnancy, which may help placental hormones become the main driving factor to regulate maternal lipid metabolism during pregnancy [53]. However, there was no significant difference in circulating concentrations of ANGPTL4 [52]. The exact mechanism by which TH upregulates ANGPTL3 remains to be further investigated.

### 1.5. Dyslipidemia May Be Involved in the Occurrence of Thyroid Dysfunction

Studies conducted on nonpregnant individuals have revealed that dyslipidemia could also contribute to the development of thyroid dysfunction. Wang et al. reported that elevated baseline TC levels were a risk factor for the progression of SCH. Furthermore, the administration of statins to reduce TC levels was associated with the restoration of normal thyroid function in SCH patients [54]. Metabolomic mass spectrometry revealed changes in the levels of lipid and lipid-related compounds (free fatty acids, polyunsaturated fatty acids, acylcarnitine, and lysophospholipids) which could be used as biomarkers indicating tissue thyroid hormone status [55]. Shao et al. reported that, in comparison to that in healthy individuals, the plasma glycerophospholipid level was significantly increased in both SCH patients and CH patients. However, there were no significant differences in metabolites between the CH and SCH groups [22]. These findings suggest that lipid disturbances may precede or accompany thyroid dysfunction and that managing dyslipidemia could mitigate the progression or severity of thyroid conditions. The relationship between dyslipidemia and thyroid function is complex and likely involves bidirectional interactions.

#### 1.5.1. Autoimmunity

One possible explanation is that dyslipidemia in patients with thyroid dysfunction may be related to thyroid autoimmunity. Compared with those in healthy individuals, the lipid metabolite patterns in euthyroid patients with Hashimoto thyroiditis (HT) were similar to those in patients with hypothyroidism [56]. This observation implies that thyroid hormones are not the only determinant of serum metabolite variations and indicates a potential role for thyroid autoimmunity in these changes. Lipid metabolism disorders are key regulators of T cell differentiation or T cell responses through transcriptional, epigenetic, or posttranslational regulation via intracellular signaling or microenvironmental factors [57]. It was reported that lipid molecules and their metabolites may participate in the occurrence and development of autoimmune diseases by regulating the differentiation of CD4+ T cells [58]. For example, certain lipids can act as ligands for T cell receptors (TCRs) on helper T cells, leading to the activation of specific immune responses. Upon engagement of the TCRs, phospholipase C (PLC) hydrolyzes phosphatidylinositol 4,5-bisphosphate (PIP2) to produce inositol trisphosphate (IP3) and diacylglycerol (DAG). IP3 serves as a signaling molecule that triggers the release of calcium from intracellular stores, and DAG activates protein kinase C (PKC). These signaling events are pivotal for the activation of transcription factors such as nuclear factor of activated T cells (NFAT) and nuclear factor-kappa B (NF-*κ*B), which are crucial for T cell function. The DAG-dependent signaling cascade is ultimately terminated by diacylglycerol kinase (DGK), which converts DAG to phosphatidic acid (PA) [59].

The balance between Th17 and Treg cells is essential for preserving immune homeostasis, with Tregs helping to suppress the activity of Th17 cells and maintain immune tolerance [60]. Dyslipidemia can disrupt this balance, leading to an increased risk of inflammation and autoimmune diseases. In patients with autoimmune thyroid disease, the proportion of Th17 cells in the peripheral blood is increased, the proportion of Treg cells is lower, and the Th17/Treg ratio is greater [61]. The effect of lipid metabolism on T cells is a complex and multifaceted process that can work through multiple pathways to affect the balance between Th17 and Treg cells. Various metabolites produced during lipid metabolism, including fatty acids, cholesterol, and phospholipids, exert direct influences on the activation, proliferation, and differentiation of T cells. Short-chain fatty acids potentiate the differentiation of Th17 cells in the spleen and mesenteric lymph nodes [62]. Long-chain fatty acids such as palmitate and oleate enhance Th17 cell differentiation and proliferation through the p38-MAPK pathway, thereby accelerating disease progression of experimental autoimmune encephalomyelitis in animal models of multiple sclerosis [63]. And n-3 polyunsaturated fatty acids have been shown to inhibit Th17 cell differentiation and regulate the Th17/Treg balance [64]. Moreover, a large abundance of plasma cholesterol disrupts T cell homeostasis, which mediates inflammation in hypercholesterolemia [65]. Some bile acid derivatives such as lithocholic acid derivative inhibit Th17 cell differentiation by binding to the Th17 cell-specific transcription factor retinoic acid receptor-related orphan receptor *γ*t (ROR*γ*t) [66]. In addition, the deoxycholic acid derivative induces Foxp3 expression by inhibiting foresaid X receptor transcriptional activity in dendritic cells and then enhances the ability to prime Treg cell differentiation from naive CD4+ T cells [67]. The process of lipid metabolism can also influence the differentiation of T cells. Studies in mice have shown that Th17 cell differentiation depends on acetyl-CoA carboxylase 1- (ACC1-) mediated de novo fatty acid synthesis, which produces phospholipids vital for cell membrane formation [68]. In ACC1-deficient murine models, the Th17 immune response, proliferation, and infiltration in colitis are suppressed, and a high-fat diet stimulates Th17 cell differentiation [69, 70]. In type 2 diabetes mellitus, inhibiting fatty acid *β*-oxidation (FAO) reduces IL-17 production by proinflammatory Th17 cells, indicating that FAO may induce insulin resistance and islet *β*-cell dysfunction through Th17 inflammation. FAO also appears to modulate the Th17/Treg balance through adenosine 5′-monophosphate-activated protein kinase (AMPK) signaling [71].

#### 1.5.2. Lipotoxicity

The thyroid might be a target organ affected by lipotoxicity. Excessive accumulation of lipids, particularly TGs, can result in steatosis in nonadipose tissues. In obese individuals, steatosis can cause cellular dysfunction in nonadipose tissues, including the thyroid, and endocrine dysfunction of thyroid follicular cells may be attributed to this condition [72]. In a case-control study of 24,100 participants, hypertriglyceridemia was significantly associated with an increased risk of hypothyroidism [20]. And another cross-sectional study found that TSH was positively associated with BMI, waist circumference, and TG concentration [73]. The results of these epidemiological studies support the possibility that obesity may affect thyroid function through lipotoxicity and contribute to the development of hypothyroidism. The same findings have been found in animal studies. In rats fed a high-fat diet (HFD), both an elevated TG content in thyroid tissue and ultrastructural modifications in histiocytic cells were observed [74]. Enhanced endoplasmic reticulum (ER) stress appears to play a significant role in the onset of thyroid dysfunction. A recent study revealed that ER stress markers were activated in the thyroid glands of rats fed a HFD and in human primary thyroid cells treated with palmitate (PA) [75]. Furthermore, swelling of the ER cavity was detected, indicating a disruption in ER homeostasis. This disturbance likely interferes with the folding and retention of thyroglobulin (Tg) within the ER, resulting in decreased Tg levels. Interestingly, cessation of the HFD and alleviation of ER stress were found to restore Tg levels and improve thyroid function. These findings suggest that ER stress is implicated in the reduction of Tg levels and the development of hypothyroidism in rats fed a HFD [76]. And under ER stress, the expression of TSHR and signaling of TSH/TSHR were reduced [77]. Besides the impact on the thyroid gland, an animal study reported that the content of cholesterol is also significantly increased in the pituitary gland of rats fed with HFD. Meanwhile, increased cell size and number of granules of TSH are found in the anterior pituitary of high-cholesterol-diet-fed rats, affecting thyroid function through the pituitary-thyroid axis [78].

#### 1.5.3. Inflammation

It is well known that adipose tissue generates and secretes a wide variety of secretory factors, including adipokines (such as leptin, adiponectin, and resistin), cytokines, and chemokines. Leptin is directly proportional to adipose tissue mass and exhibited higher levels in most obese patients which plays a crucial role in regulating the production of neurohormones in the mediobasal hypothalamus, including thyrotropin-releasing hormone (TRH) neurons of the periventricular nucleus [79]. Leptin receptors, which directly suppress TSH secretion and the expression of the sodium-iodide symporter (NIS) and thyroglobulin mRNA in thyroid cells, have been identified in the anterior pituitary and thyroid gland [80]. Tomov et al. found that serum levels of leptin in HT patients were higher than those in the healthy group, and the CH group showed significantly higher levels of leptin than those of the controls with normal thyroid function [79, 81]. Chronic low-grade inflammation caused by cytokines secreted by adipose tissue could also affect thyroid function. Yang et al. found upregulation of proinflammatory cytokine gene expression interleukin 6 (IL-6), interleukin 1*β* (IL-1*β*), and tumor necrosis factor *α* (TNF-*α*) in HFD mice and significantly decreased anti-inflammation cytokine IL-10 mRNA expression levels in the thyroid, suggesting an enhanced thyroid inflammatory response [82]. An increase in IL-6, IL-1*β*, and TNF-*α* can inhibit the expression of NIS mRNA and influence iodide uptake activity. Moreover, these cytokines may contribute to morphological changes in the thyroid [83]. In addition, chronic inflammation might also affect thyroid function by modulating deiodinase [84].

### 1.6. Dyslipidemia and Thyroid Disease in Early Pregnancy and Adverse Pregnancy Outcomes

Clinical or subclinical hypothyroidism during pregnancy is associated with dyslipidemia, which is characterized by elevated levels of TC, TGs, and LDL-c. These lipid abnormalities can have implications for glucose metabolism and increase the risk of gestational diabetes mellitus (GDM) and other complications (as shown in Table 1). Sobia et al. reported that high levels of TG, TC, LDL-c, and VLDL in the first trimester can be considered as strong predictors of GDM in the last trimester, and low levels of HDL-c in the first trimester can also be used as indicators of GDM in the last trimester [85]. Numerous studies found a strong positive correlation between TG levels in early pregnancy were the strongest predictors of GDM [86, 87]. Wang et al. identified 10 lipid biomarkers in the first trimester that were significantly associated with the risk of GDM, with some lipids showing positive associations and others showing negative associations [88]. Dyslipidemia in pregnant women may contribute to an increased risk of preeclampsia and other complications [89]. Yelverton et al. found that increased maternal TG levels during early pregnancy were associated with faster infant weight gain at 20–34 weeks of gestation [61]. Adank et al. reported that early pregnancy TG and remnant cholesterol levels were associated with an increased risk of large for gestational age (LGA), although these associations were not significant after adjustment for glucose [90]. Lipidomic studies by Li et al. showed that newborn birth weight was positively correlated with certain lipid metabolites such as SM or PE levels [24]. These altered lipid metabolites could be used as early predictors of adverse maternal and neonatal outcomes [24].

### 1.7. The Current Methods Targeting Dyslipidemia in Pregnant Women with Thyroid Disease

For pregnant patients with hypothyroidism, clinical guidelines suggested that L-T4 replacement therapy should be given promptly to mitigate the risk of dyslipidemia and adverse neonatal outcomes [14]. Although observational studies have demonstrated that dyslipidemia has also been observed in SCH pregnant women [18–24]. A large cohort study involving 20,365 participants demonstrated that L-T4 treatment significantly reduced TC and LDL-c levels in SCH patients during the first trimester [91]. This indicates that thyroid hormone replacement contributes to the normalization of lipid profiles in pregnant women with CH or SCH. However, uncertainty persists regarding the association about L-T4 replacement, modifying lipid metabolism and adverse pregnancy outcomes in pregnant women with SCH [25, 26]. Further research is required to determine the treatment goal and timing of L-T4 therapy in SCH pregnant women.

The current use of other lipid-lowering agents for pregnancy is constrained and limited [91–95]. Nonpharmacological lifestyle changes remain to be the primary management for hyperlipidemia in pregnant women. The adoption of a healthy lifestyle includes a diet low in fat and cholesterol, alongside increased physical activity [96]. Studies by Wang et al. and Farias et al. have highlighted the importance of prepregnancy BMI in predicting the occurrence of hypertensive diseases and changes in lipid levels during pregnancy, underscoring the significance of proactive intervention before or in the early stages of pregnancy [97]. Additionally, omega-3 fatty acids supplementation during pregnancy has been shown to reduce TG by 20-30% and slightly lower non-HDL-c and Apo-B levels, while also reducing the risk of preterm birth and perinatal death [94].

## 2. Conclusion

In conclusion, our review has summarized the complex relationship between lipid metabolism and thyroid dysfunction in pregnant women. Pregnant women with clinical or subclinical hypothyroidism during pregnancy are at a greater risk of dyslipidemia, which can subsequently increase the incidence of adverse pregnancy outcomes. The mechanism by which thyroid function regulates lipid metabolism during pregnancy and its relationship with changes in lipid profiles during the development of adverse pregnancy outcomes need to be further studied. Furthermore, understanding the complex relationships among thyroid function, lipid metabolism, and pregnancy may lead to improved diagnostic and therapeutic strategies for managing thyroid disorders during pregnancy. Whether LT4 therapy can provide lipid benefits in pregnant women with subclinical hypothyroidism and which patients will benefit the most also need to be confirmed in well-designed prospective studies.

## Figures and Tables

**Table 1:** Literature review in lipidomic study of pregnant women.

Authors	Participants	Gestational week	Thyroid status	Ethnicity	Key points
Liu et al. [15]	1650 pregnant women	In the first trimester	CH and SCH	Chinese	Patients with clinical hypothyroidism exhibited higher levels of Apo-A1 and Apo-B compared to those with normal thyroid function. TC was positively correlated with TSH and negatively correlated with FT4. The SCH group had a higher TC and LDL-c level than the euthyroidism group
Xu et al. [16]	152 patients with hypothyroidism during pregnancy	In the second trimester	CH, SCH, and hypothyroxinemia	Chinese	The fasting blood glucose and HbA1c, TC, and LDL-c of the CH group and the SCH group were higher than the low T4 group and the normal group, and the CH group was higher than the SCH group.
Han et al. [19]	14123 pregnant women	From the second trimester until delivery	SCH	Chinese	In the second and third trimesters, HDL-c and LDL-c in the SCH patients treated with levothyroxine were significantly lower than that in the control group. And glycometabolism was significantly different between the control group and pregnant women with SCH even after levothyroxine treatment.
Zhang et al. [21]	240 pregnant women	11–14th, 22–28th, and 32–34th weeks of gestation	SCH	Chinese	Fatty acids in the first trimester did not significantly distinguish between pregnant women with SCH and normal groups. Levels of three fatty acids in the second trimester and two fatty acids in the third trimester were significantly increased in SCH group
Cai et al. [23]	55 pregnant women	In the third trimester	SCH	Chinese	Increased levels of PC (36 : 2), PE (36 : 4) and decreased levels of SM (d42:6), SM (d42:7), etc can be used as characteristic metabolites for patients with hypothyroidism during pregnancy.
Li et al. [24]	60 pregnant women	In the third trimester	SCH	Chinese	SM and PE levels were higher in the subclinical hypothyroidism group compared to controls. Birth weight was lower compared to controls.
Yelverton et al. [61]	284 pregnant women	In the first trimester	Euthyroidism	Irish	Increased maternal TG levels during early pregnancy were associated with faster infant weight gain at 20-34 weeks of gestation and slower abdominal circumference growth at 2-5 years postpartum.
Sobia et al. [85]	300 pregnant women, 176 with positive and 124 with negative familial background of GDM	In the first trimester	Euthyroidism	South Asian	High levels of TG, TC, LDL-c, and VLDL in the first trimester can be considered as strong predictors of GDM in the last trimester, and low levels of HDL-c in the first trimester can also be used as indicators of GDM in the last trimester.
Lu et al. [86]	16489 healthy singlet pregnant women	In the first trimester	Euthyroidism	Chinese	TC, TG, and LDL-c were the most effective indicators for predicting the development of PE, and the elevated levels of TC and TG were risk factors for PPH during the first trimester.
Sweeting et al. [87]	980 pregnant women	In the first trimester	Euthyroidism	Caucasian, East Asian, South Asian, and others	TG during the first trimester, independent of BMI, were the strongest lipid/adipokine predictor for GDM.
Wang et al. [88]	7407 pregnant women	In the first trimester	Euthyroidism	Chinese	10 lipid biomarkers in early pregnancy were significantly associated with GDM risk. Positive associations were found for PC (O-36 : 1), PE (O40:5), PE (P-38 : 6), PI 40 : 6, and DG 18 : 0/18 : 1; while negative associations were found for MHC 18 : 0, DHC 24 : 0, DHC 24 : 1, SM 34 : 1, and PC 40 : 7.
Adank et al. [90]	5702 women with a live-born singleton	In the first trimester	Euthyroidism	European	TG and remnant cholesterol levels in early pregnancy were positively associated with the risk of large for gestational age (LGA), independent of maternal BMI.

## Data Availability

All data used to support the findings of this study are available from the corresponding author upon request.

## Abbreviations

TH: Thyroid hormone
TGs: Triglycerides
HL: Hepatic lipase
CETP: Cholesterol transfer protein
HDL-c: High-density lipoprotein cholesterol
LDL-c: Low-density lipoprotein cholesterol
VLDL: Very low-density lipoprotein
Apo-B: Apolipoprotein B
TC: Total cholesterol
TSH: Thyroid-stimulating hormone
FT4: Free thyroxine
Apo-A1: Apolipoprotein A1
SCH: Subclinical hypothyroidism
LC–MS: Liquid chromatography–mass spectrometry
CH: Clinical hypothyroidism
LPC: Lysophosphatidylcholine
PC: Phosphatidylcholine
PE: Phosphatidylethanolamine
SM: Sphingomyelin
mRNA: Messenger RNA
TSHR: Thyroid-stimulating hormone receptor
HMGCR: Hydroxy methylglutaryl coenzyme A reductase
FFA: Free fatty acid
SREBP: Sterol regulatory element binding protein
LDLR: LDL receptors
ChREBP: Carbohydrate response element-binding protein
FGF: Fibroblast growth factor
WAT: White adipose tissue
PCSK9: Proprotein convertase subtilisin 9
ANGPTLs: Angiogenin-like proteins
TCRs: T-cell receptors
PLC: Phospholipase C
PIP2: Phosphatidylinositol 4, 5-bisphosphate
IP3: Inositol trisphosphate
DAG: Diacylglycerol
PKC: Protein kinase C
NFAT: Nuclear factor of activated T cells
NF-*κ*B: Nuclear factor-kappa B
DGK: Diacylglycerol kinase
PA: Phosphatidic acid
ACC1: Acetyl-CoA carboxylase 1
FAO: Fatty acid *β*-oxidation
AMPK: Adenosine 5′-monophosphate-activated protein kinase
HFD: High-fat diet
ER: Endoplasmic reticulum
Tg: Thyroglobulin
TRH: Thyrotropin-releasing hormone
HT: Hashimoto thyroiditis
NIS: Sodium-iodide symporter
IL-1: Interleukin 1
IL-6: Interleukin 6
GDM: Gestational diabetes mellitus
FBG: Fasting blood glucose
LGA: Large for gestational age
L-T4: Levothyroxine.
